# Predictors and Outcome of Ventriculoperitoneal Shunt Infection: A Retrospective Single-Center Study

**DOI:** 10.7759/cureus.27494

**Published:** 2022-07-30

**Authors:** Maria Abuhadi, Reema Alghoribi, Lama A Alharbi, Zahrah Barnawi, Raghad AlQulayti, Arwa Ahmed, Maha Al-Alawi, Saleh S Baeesa

**Affiliations:** 1 Division of Neurosurgery, King Abdulaziz University Faculty of Medicine, Jeddah, SAU; 2 Division of Infectious Diseases, King Abdulaziz University Faculty of Medicine, Jeddah, SAU; 3 Neurosciences, King Faisal Specialist Hospital and Research Centre, Jeddah, SAU

**Keywords:** shunt infections, risk-factors, csf infection, ventriculoperitoneal shunt complications, hydrocephalus management

## Abstract

Background:Shunt infection critically affects approximately 8-10% of all inserted shunts, leading to significant morbidity and mortality. This study aimed to assess the clinical and laboratory factors associated with shunt infection and outcomes in patients treated for hydrocephalus.

Methods*: *A retrospective study was performed on patients who underwent ventriculoperitoneal shunt (VPS) surgery for hydrocephalus between January 2015 and June 2018. The primary outcome was the development of shunt infection following VPS surgery. Records were reviewed, and variables were analyzed, including patients' demographics, perioperative laboratory and shunt data, and outcomes. The patients had five years of follow-up from surgery, including a minimum of two years from the onset of VPS infection.

Results:A total of 132 shunts were inserted in 103 patients with a mean age of 2 years (range; 2 days to 73 years), and 53.4% were males. Twenty-two patients were suspected of having VPS infection (16.7% per procedure); only six (4.5%) had positive cerebrospinal fluid (CSF) detected organisms. Patients with preoperative hemoglobin, white blood cells, and serum glucose within normal values had a lower shunt infection rate. The pediatric population had an elevated risk of VPS infection, particularly those who underwent surgery at a younger age than 7.5 months, weighed less than 10 Kg, and were associated with myelomeningocele. in addition, a shorter surgery time of less than 82 min, single surgeon, and operating room of fewer than four attendees are associated with lower risk of VPS infection.

Conclusion:We emphasize that early identification and modifications of the risk factors can minimize the probability of developing VPS infection and improve patients outcome.

## Introduction

Hydrocephalus is a condition in which there is a build-up in the cerebrospinal fluid (CSF) either due to overproduction of CSF or decreased absorption, causing dilation of the ventricles. This accumulation is treated with CSF diversion, shunt, which has been considered the mainstay and adequate treatment of hydrocephalus since the 1950s for an estimated 40,000 shunt placements annually in the USA [1].

However, a revision of about 14,000 shunts per year is done due to the high rates of shunt complications, which have significant patient morbidity and an enormous financial burden on health care systems. Furthermore, like any procedure of foreign body implantation, infection and mechanical dysfunction are expected and can have severe consequences [2,3].

Since the implementation of VPS over the last 60 years, infection critically affects approximately 8-10% of all shunted patients, leading to significant morbidity and mortality and generally occurring a few months following surgery [4,5]. The most common pathogens reported causing shunts infection are coagulase-negative staphylococci, especially *Staphylococcus epidermidis*, located on the surface of a person's skin and in the sweat glands and hair follicles deep within the skin, followed by *Staphylococcus aureus* and a variety of gram-negative rods [6-8].

Several risk factors were highlighted in the literature; age at VPS placement is one of the most critical factors of infection. Insertion of VPS in premature patients under one year due to poor immunity and the immature skin barrier is associated with a higher rate of infection [6]. Also, the length of the procedure is considered another risk factor since most VPS infections occur in the perioperative period [9]. Patients who had multiple shunt revisions, previously infected shunts, used endoscopic ventricular procedures, and presented with a postoperative surgical site CSF leak are also recognized as risk factors [6,7,10].

There are ongoing demands from several studies for determining the predictors of VPS infection to modify them, lower their impact on morbidity and mortality, and improve patients’ outcomes. Similarly, our study aims to evaluate predictors and outcomes of VPS infection from a single center with a five-year follow-up.

## Materials and methods

Study design

We performed a retrospective review of patients with hydrocephalus who underwent VPS surgery, either insertion or revision, following between January 2015 and June 2018 at King Abdulaziz University Hospital (KAUH), Jeddah, Saudi Arabia. In addition, at least a two-year follow-up was obtained from the last shunt or complication. The primary outcome of this analysis was the number of shunt infections following VPS surgery. We excluded other than peritoneal shunt surgeries such as atrial or pleural placements.

This study was approved by the unit of the Biomedical Ethics Committee of the Faculty of Medicine, King Abdulaziz University (Reference No. 148-18), and the requirements for gaining informed consent from patients were waived due to the retrospective nature of the research.

Data collection and inclusion criteria

Patients’ electronic records were reviewed from the Phoenix system of KAUH using a data collection sheet via Google forms. The data included patients' demographics, medical history, the etiology of hydrocephalus, and history of previous shunt insertions. In addition, perioperative data had a date of admission to surgery time, length of stay, timing during the day and urgency of the procedure, duration of surgery, antibiotic prophylaxis, and the number of scrubbed and attended staff in surgery.

Medical records of preoperative laboratory workups such as hemoglobin (Hb), white blood cell (WBC), platelets (Plt), serum glucose, and albumin (Alb) were analyzed. In addition, laboratory work parameters were categorized into groups based on what our hospital uses commonly as cut-offs. Hb, WBC, and Plt levels were evaluated for 129 cases, Alb for 78 patients, and glucose for 86 cases. We excluded those tests which exceeded the reasonable period as per the guidelines of the American Society of Anesthesiologists, which stated that six months is an adequate time to rely on preoperative tests if the patient's medical history did not change [11].

Diagnosis of shunt infection

The time from surgery, clinical, surgical site and systemic presentations, and CSF analysis and culture were recorded in suspected VPS infection cases. Patients were categorized into three groups, as summarized in Table 1.

**Table 1:** Categorization of shunt infection in the study.

Groups	Category	Systemic symptoms and signs	Surgical site symptoms and signs	CSF analysis/Culture
1	Definite	Present	Present	Pleocytosis, high protein and low sugar. Pathogens isolated.
2	Probable	Present	Present, mild	Pleocytosis, high protein and low sugar. No pathogens isolated.
3	Suspicious	Present, mild	Absent	Not performed

Statistical analysis

Data entry was done by the Microsoft excel program and analyzed using the SPSS software package version 21.0 (IBM Corp., Armonk, NY). Descriptive statistics for patient characteristics, the etiology of hydrocephalus, shunt operations, and revisions. Kaplan-Meier estimates were used to analyze the time to shunt infection. Univariate analysis was conducted on all types of data. Bivariate analysis with a p-value of 0.05 was applied to detect the significance. A Chi-square test assessed risk factors like age at first insertion and preoperative use of antibiotics prophylaxis concerning complication development. One-way ANOVA was used to study the relationship between the duration from surgery date to the onset of complications and the patient's age at surgery.

## Results

Patients’ characteristics

A total of 103 patients with a mean age of two years (range; 2 days to 73 years) who underwent 132 VPS procedures were included in the study and summarized in Table 2.

**Table 2:** Patients’ demographics.

Number	103 Patients (123 procedures)	
Total	Children (15 years or less of age ) (n= 77)	Adult (n= 26)
Mean age of children (months)	28.7	
Mean age of adults (years)	41.04	
Sex, n (%)		
Male	55 (53.4)	
Female	48 (46.6)	
Diagnosis, n (%)		
CNS tumors	18 (17.5)	
Meningomyelocele	15 (14.6)	
Intraventricular hemorrhage (IVH)	11(10.7)	
Post-infection (meningitis, encephalitis)	6 (5.8)	
Dandy-Walker syndrome	3 (2.9)	

The majority were children (74.8%) with a mean age at surgery of 28.7 months (range; 2 days-14 years), and 26 were adults (25.2%) with a median of 47.5 years (range; 15- 73 years). Male to female ratio was 1.14:1, and 55 (53.4%) patients were males. The causes included CNS tumor (17.5%), meningomyelocele (14.6%), post-infection (meningitis, encephalitis) (5.8%), intraventricular hemorrhage (IVH) (10.7%), and Dandy-Walker syndrome (2.9%). Meningomyelocele was more prevalent in females (12; 25%), while CNS tumors were males' most common etiological factor (8; 14.5%).

Overall shunt complications

**Figure 1:**
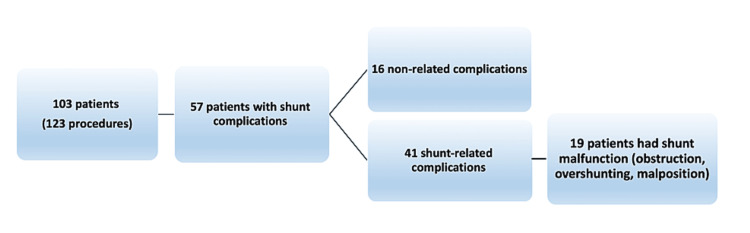
Algorithm showing the clinical course of patients presenting with shunt complications.

Fifty-seven cases developed postoperative complications following VPS with a mean onset time of 116 days (range; 0- 690). Nineteen patients (33.33%) developed complications during the same admission, while 38 (66.67%) were readmitted after presenting to the emergency department in the mean of 3.5 months (range; 7 days to 23 months). Forty-one (33.3%) out of 123 VPS procedures were VPS-related complications; 19 (15.4%) were related to shunt malfunction not associated with infection, while the remaining 16 (13%) were not associated with VPS surgery like upper respiratory tract infections (Figure 1).

Radiological investigations for all patients with VPS-related complications included an emergency brain CT scan; 14/57 (30.4%) showed an interval increase of ventricular size or fixed ventricular size after placement of the shunt. In addition, an abdominal ultrasounds scan was performed for 10 cases; two patients had abdominal pseudocyst formation.

Laboratory workups for all patients with postoperative VPS complications included a complete blood count (CBC) to look for abnormal WBCs, C-reactive protein (CRP), and CSF analysis and culture, the most important diagnostic test. The latter was done for 24 out of 57 concerned cases, where it showed abnormal CSF leukocyte count (>5 cells/mm^3^) in 21 out of 24 (87.5%) cases.

Shunt infection

A diagnosis of VPS infection was determined by suggestive surgical systemic and site clinical manifestations of infection, abnormal laboratory findings of CSF parameters, and/or growth of a pathogen in CSF culture. The clinical symptoms and signs at the time of the presentation. Fever (n=19; 86.4%) and vomiting (n=10; 45.5%) were the most common presentation upon readmission (Table 3).

**Table 3:** Clinical characteristics of shunt-associated infection

Variable	Number of cases
Fever (>38^0 ^C)	19 (86.4%)
Vomiting	10 (45.5%)
Surgical site inflammatory changes	7 (31.8%)
Decreased oral activity	7 (31.8%)
Irritability	6 (27.3%)
Decreased activity	5 (22.7%)

**Figure 2:**
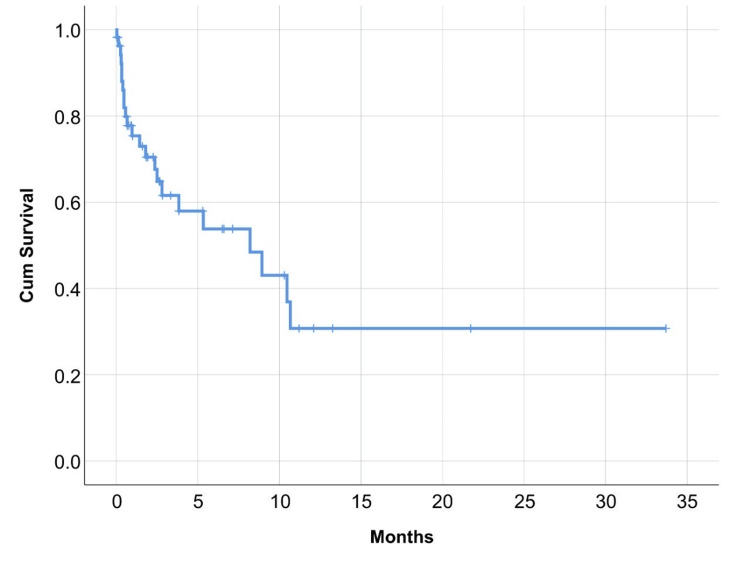
Kaplan-Meier estimate for median (95%CI) time to infection onset was 8.2 months (2.1-14.3).

Shunt infection was suspected in 22/123, with an incidence rate of 16.7% per procedure with a median (95% CI, 2.1-14.3) time to infection onset was 8.2 months (Figure 2).

Twelve patients (54.4%) had delayed presentation in a period between 1 to 12 months, while nine (40.9%) manifested earlier (within a month; with the earliest of one day following surgery). One patient presented late at 23 months from surgery.

CBC was done for 54 cases with WBCs mean of 14.2 K/uL (range, 3.3- 40.75 K/uL), where a count of >12 K/uL was noticed in 31 cases (57.4%). CRP level was >3 mg/L in 27 out of 39 cases (69.2%) with a mean of 46.2 mg/L (range, <3- 221 mg/L).

CSF parameters in 17/22 with suspected VPS infection revealed a cell count median of 125 cells/mm^3^ (range, 13- 11000 cells/mm^3^), glucose mean of 1.96 mmol/L (range, 0.1- 4.1 mmol/L), and proteins median of 3.02 g/dl (range, 0.12- 22 g/dl).

A definite VPS infection was identified in 6/22 (4.9% of 123 procedures) with positive CSF culture results for the following pathogens: (*Staphylococcus aureus*, *Pseudomonas aeruginosa*, *Escherichia coli*, and *Acinetobacter baumannii*). Besides, a polymerase chain reaction (PCR) test was done for one patient revealing Human Herpesvirus 6 (HHV-6). Nine patients had clinical presentations of VPS infection with abnormal CSF analysis but negative CS. The third category of suspected VPS infection had seven patients with mild clinical manifestations treated with empirical antimicrobials at a meningitis dose without clearly identifying a laboratory or CSF abnormality. Vancomycin was used in all cases in addition to another agent (ceftazidime in eight cases, ceftriaxone in five, meropenem in two, or cefotaxime in one), with a median duration of 14 days (range, 7 to 28 days).

**Figure 3:**
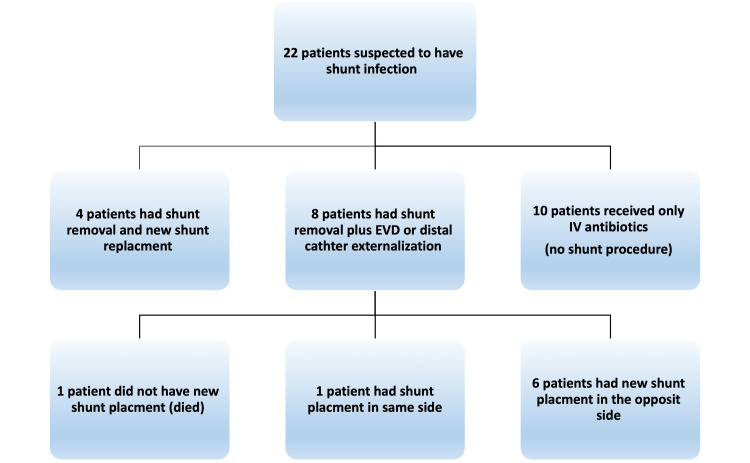
Algorithm showing the clinical course and management of patients presenting with a shunt infection.

The initial management of VPS infection involved the administration of intravenous antibiotics in all patients following CSF sampling or empirically. Patients with suspected infection (10 out of 22) with negative CSF cultures had immediate resolved symptoms in 24 hours and were treated for 10 days with intravenous antibiotics only, without surgical intervention. In the remaining 12 patients, surgical interventions were performed as a one-stage surgery with removal of infected VPS and insertion in the opposite side of a new VPS in four patients. While a two-stage VPS replacement in eight cases; consisted of complete shunt removal followed by external ventricular drain (EVD) insertion in seven patients or distal end shunt externalization in one patient. Once at least two consecutive CSF cultures occasions were obtained, a new VPS was placed on the opposite side in nine patients; one patient had VPS placed on the same side. The antibiotics were used for a mean of three days (range, 1-5) after the new VPS placement (Figure 3).

This study revealed one mortality (1%); one child presented with acute hydrocephalus with probable VPS infection with a Glasgow coma score of 4. He underwent EVD and VPS removal, and his CSF culture was negative. Unfortunately, the patient did not recover and died eight days after admission. 

During the three-year study period and two-year follow-up from the episode of VPS infection, of the 12 patients treated with antibiotics and VPS replacement, only one patient presented with a relapse of shunt infection within four months that required subsequent VPS replacement. In addition, three out of 10 patients treated with intravenous antibiotics only presented later with VPS infection within six months, requiring subsequent VPS replacement. However, no relevant association was observed between the outcome of the VPS infection treatment methods and infection relapse or among patients with CSF culture status (p= 0.9).

Patients’ characteristics analysis

**Figure 4:**
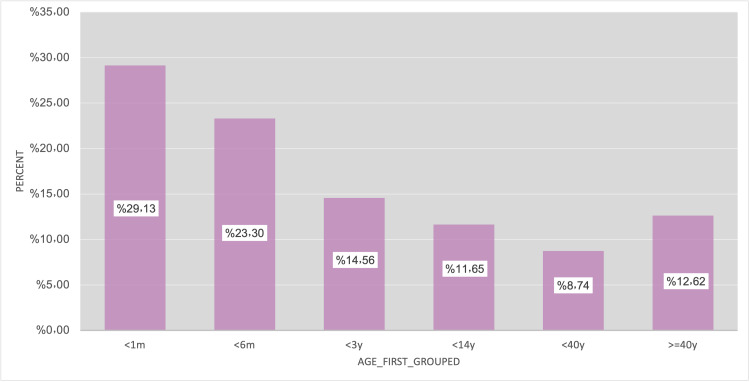
Age of patients at their first-time shunt insertion.

This study analyzed age at first VPS insertion, which showed that 54 patients (52.4%) had their first insertion when they were younger than six months of age. A chi-square test was done to detect if age at surgery contributes to a risk factor for shunt infection (Figure 4). Pediatric patients' age in which they had their first insertion had a mean of 7.5 months; they presented later with shunt infection compared to those older than a mean of 20.5 months (p= 0.039).

The patients’ weight at surgery was studied, with a mean weight of 7.95 kilograms (range, 1.15-103.3 kg). After grouping patients into adults and pediatrics (younger than 15 years of age), a significant correlation was found between developing shunt infection and body weight in the pediatric age group (p= 0.015). Patients who developed shunt infection (range: 1.15-66 kg) had a weighted mean of 6.3 kg, with 94.1% weighing 10 kgs and less. While those who did not have shunt infection, a mean of 10.2 kg, including all cases, were weighing more than 20 kgs, and 89.5% between 10-20 kgs.

The relationship between developing VPS infection and etiology of hydrocephalus showed a significant difference (p= 0.032), as 31.7% of the cases that developed infection had myelomeningocele, followed by Dandy-Walker syndrome (14.6%) and CNS tumor (14.6%).

Perioperative clinical factors analysis

Eighty-four VPS were inserted for the first time (63.6%), while 48 were revisions (36.4%), which included 17 ventricular catheters, seven peritoneal catheters, and 24 total VPS replacements). A significant relationship showed that most cases (75.8%) did not result in shunt infection, and those had no previous shunt surgery (p= 0.002).

Urgency and timing of surgery were analyzed, reflecting whether the quality of preoperative preparation and the physicians' workload can predict the risk of VPS infection. Eighty-seven VPS operations (66%) were done emergently, and 45 electively (34%). Surgeries during the daytime were categorized into four groups; in the morning (8-11:59 am): 64 (48.5%), in the afternoon (12-4:59 pm): 40 (30.3%), in the evening (5-11:59 pm): 19 (14.4%), at night (12-7:59 am): 9 (6.8%). The study showed no statistical significance regarding the urgency, timing of surgery during day or night, or length of hospital stay before the surgery; the latter has a mean of about 13 days (range; 0- 109 days).

The median duration of all VPS operations was 73.5 minutes, with a minimum of 16 minutes and a maximum of 3 hours and 56 min. A cut-point of 82 minutes duration of surgery shows a difference, whether leading to shunting infection (p=0.043).

The total number of scrubbed surgeons and staff in the operating room ranges between 1-3 and 3-10, respectively. The analysis revealed that 77.3% of surgeries were performed by one surgeon, and they have not developed a shunt infection (p= 0.04). On the other hand, operating room attendees of more than four (range, 5-10) in the operating room demonstrated a significant difference in a shunt infection (p= 0.007) noticed only in the adult age group (Table 4).

**Table 4:** Attendees in the operating room as a risk factor for developing VPS infection Note: attendees demonstrated a significant difference (p= 0.007), noticed only in the adults' age group.

Age	Attendees number during shunt surgery	Infected	No Infection	Total
Pediatric	Attendees 1-4	6 (31.6%)	13 (68.4%)	19
	Attendees > 4	10 (12.2%)	72 (87.8%)	82
	Total	16	85	101
Adults	Attendees 1-4	2 (8%)	23 (92%)	25
	Attendees > 4	4 (66.6%)	2 (33.3%)	6
	Total	6	25	31

Regarding preoperative antibiotics prophylaxis, 83 cases were given (63%), whereas the most frequent one was cephalosporin, which was used in 78 surgeries (94%). The remaining five cases were given vancomycin (2.4%), cefuroxime (2.4%), and cefotaxime (1.2%). There was a statistically significant relationship between the administration of preoperative antibiotic prophylaxis and VPS infection (p= 0.024).

Preoperative laboratory tests analysis

Laboratory investigations before shunt surgery were studied within six months, including complete blood count (hemoglobin, white blood cells, platelets), random serum glucose, and albumin, to detect any correlation with developing complications or infection. One hundred thirty cases had preoperative medical records of hemoglobin, white blood cells, and platelets with a mean of 11.8 mg/dl, 11 K/uL, and 445.4 K/uL, respectively. In addition, albumin was evaluated in 77 cases with a mean value of 31.7 g/L, and serum glucose for 83 patients had a mean of 5.9 mmol/L.

Patients with normal hemoglobin levels (67.1%) did not develop shunt infection. However, this correlation was significant only in the pediatric age group (p= 0.043) but not among adults (p= 0.71).

Another significant association between preoperative serum glucose and WBC levels was found with a shunt infection; 88.2% of non-infected shunts had a normal preoperative WBCs level (p= 0.032). However, 60% of cases with a normal preoperative glucose level did not develop shunt infection (p= 0.037). Albumin levels and platelet counts were not found to be significant risk factors.

## Discussion

Cerebrospinal fluid shunts for treating patients with hydrocephalus demonstrated conspicuous patient outcome improvement. However, its related shunt complications, particularly infection, remain a critical cause of morbidity and mortality.

An early report by Borgbjerg et al. reported a low shunt infection rate of 2.6%; however, this low percentage was not due to an actual reduction in the infection rate but because of poor detection and suboptimal diagnosis [12]. Since then, the incidence has increased with the increased use of CSF sampling diagnostic modalities to distinguish shunt infection diagnosis from other shunt pathologies [13].

In our study, Infections occurred in 22 cases following 132 procedures, with an overall rate of 17.5 per patient. Per procedure, the infection rate of 16.7% accords with another literature rate, which ranges from 8.5 to 15% despite half of the patients considered with shunt infection had minimal clinical evidence and no CSF indicators of infection [9,14]. As expected, the infection rate per procedure is higher than the infection rate per patient since one patient can have multiple shunt placements or revisions [15].

Somehow this wide variability is explained by each report's definition of shunt infection, duration of surveillance, and/ or different study designs [7,11,15,16]. While it is not well known how the shunt infection develops, several elements are thought to contribute, of which age is considered the most significant patient factor. Although another study identified many factors, they are still not considered a standard because of unclear agreements among data [15].

In some retrospective studies, the primary cause of hydrocephalus in patients with shunt placement is intraventricular hemorrhage (IVH) [6]. However, it constitutes only 10.7% of our patients compared with our results. On the other hand, the primary cause in our patients is CNS tumors, with more prevalence among male patients (14.5%). Similar results in a study by Lee et al. reported the incidence and risk factors of VPS infection in the pediatric department are measured, showing that approximately 77% of males (152 out of 198) had CNS tumors [9]. However, a previous study revealed no significant correlation between the etiology of hydrocephalus and infection rate [17].

In our study, a significant relationship (p= 0.039) was found between the age of patients where the shunt was inserted for the first time and getting shunt infection, with most of the patients falling under the age category of an age of less than one month 46.6%. Similar results were seen in another study in Hafuf, Saudi Arabia, accounting for 29.13% [18].

Pople et al. stated that infants aged less than six months have a higher risk of infection and complications, especially in those groups who were underweight at birth or premature. This was explained by infants' poor cellular and humoral immunity, incomplete development of skin barrier, and history of other comorbidities or procedures [19].

The duration of the surgical procedure is considered a risk factor in several studies since most shunt infections occur around the perioperative period [9]. It was reported that a higher infection rate (16%) was found in patients whose shunt surgeries averaged one hour compared to operations lasting less than 30 minutes (8%) [20].

For the diagnosis of shunt infection, laboratory testing is considered the initial step as the clinical evaluation is indeterminate. Therefore, our study was determined by specific criteria, including the growth of a pathogen in CSF culture, the most precise and diagnostic for shunt infection [3]. As a result, only six cases had a positive CSF culture; however, only one (*Staphylococcus aureus*) is a gram-positive organism, while others, gram-negative organisms, include *Pseudomonas aeruginosa*, *Escherichia coli*, and *Acinetobacter baumannii*. In addition, PCR was done for one patient who revealed Human Herpesvirus 6 (HHV-6).

As well-known in clean surgical wounds, the expected pathogen to cause infection is the patient's skin flora, which, when incised, will be at risk of getting contaminated. In addition, infection is occasionally caused by an exogenous source like operating theater, tools, or instruments. However, in shunt infection, leading factors might differ due to the implantation of a foreign body, which is a risk factor for surgical site infection. This is consistent with what many believe is usually due to the spread of pathogenic organisms intraoperatively, either from contaminated skin or materials used [18]. Previous studies demonstrated that coagulase-negative Staphylococci and *S. aureus* were the most isolated pathogens [9].

In our study, the most common pathogen could not be determined. We believed that the small population of patients was responsible for the isolated cases of positive results. There is also a probability that receiving previous antibiotics therapy may play a role in negative CSF culture results. While assuming the role of prior antibiotics use, this might raise the question of whether this behavior accounts for higher infection rates since detecting and recognizing definite diagnoses is becoming more complex with negative cultures. A follow-up study is recommended to evaluate the outcomes of this study with others considering the corresponding percentage of positive cultures and subsequent management. 

The symptoms of shunt infection are variable, dependent on the type of infective organism, or maybe related to age. Some symptoms and signs suggest shunt infection like fever, or nonspecific as headache, vomiting, change in mental status, or seizures. Both fever (n=21, 16.2%) and vomiting (n=15, 11.5%) were the most common clinical symptoms in this study and other studies [7,9].

An extended hospital stay before shunt insertion increases the likelihood of infection with *S. aureus* [6]. However, like previous studies, ours doesn't show any relation between a hospital stay and getting any infection [4,10].

Effective infection prevention in the operating room is the key to reducing its rate. One of the most important methods is the administration of prophylactic antibiotics perioperatively [21]. In our study, antibiotics were used in 83 operations (62.9%), were only 78 cases (93.9%) used cephalosporin, an effective against Staphylococci, Streptococci, *Escherichia coli*, *Proteus mirabilis*, and *Klebsiella pneumonia* since it covers the most likely surgical site pathogens [22].

In a randomized clinical trial to differentiate between the effect of vancomycin and cephalosporin in hospitals and the high prevalence of methicillin-resistant *Staphylococcus aureus* (MRSA), after a four-week follow-up, 16 shunts were infected among 176 patients (9%) who were treated with cephalosporin or vancomycin immediately before surgery. It was found that the prevalence of these infections was lower among vancomycin-treated patients (4% vs. 14%). Also, a reduction in the length of hospitalization post-surgery and mortality rate was more moderate in the vancomycin group [23].

Our hospital still prefers cephalosporin because of its approved effectiveness in preventing infection. In addition, the previous year's study significantly reduces the infection rate (p= 0.001), and MRSA is not the dominant pathogen in shunt infection at our center.

Earlier studies showed a higher infection risk associated with re-inserted shunts due to previous infection [24]. Our study shows a statistical significance (p= 0.002) in similar findings where 75.8% of non-complicated shunts had no history of the previous insertion. Another study in pediatric Korean patients also showed a higher tendency for re-inserted shunts to get infected (17.2%) compared to those with first-time insertion (8.9%), however, the study lacked statistical power, so this variable was insignificant [9].

A study by Tuli et al. was done on neonates born between 2000 and 2005 who underwent shunt procedures and revealed that weight at the time of procedure was statistically different between the group with failed VP shunt versus those without failure [25]. This study also showed significance between weight at the surgery and shunts complication. However, another research revealed that patients who weighed less than 3000 g had no statistically significant difference in infection rate [26].

To be considered a protective factor, the development of shunt complications might be prevented by minimizing the number of surgeons handling the procedure. Our study upraised this, which shows that 77.3% of surgeries done by one surgeon had not developed shunt-related complications (p= 0.04). This event can be indirectly explained by the well-known mode of infection transmission, which is the increased frequency of manual contact of the shunt hardware by the surgeon's gloves [9,10]. So further prevention can be achieved by double gloving and changing gloves before dealing with the device [10,27].

As a part of behavioral factors, restricting the number of personnel and more efficiently controlling their movements in the operating theaters might reduce airborne particles, hence restraining the dispersion of bacteria [28]. In our study, determining the total number of personnel was achieved, where a statistically significant relationship with a p-value of 0.007 was found in the adults' age group (n=31). Of which, 92% (23 of 25) of procedures with ten personnel attending or less did not develop shunt infection, and two-thirds (4 of 6) of procedures with more than ten staff in the operating room developed shunt infection later. We can justify the lack of this factor in the pediatrics group by the more crucial risk factors that might outweigh this difference or because of the small adult population analyzed.

A thorough preoperative laboratory evaluation is essential to ensure patients’ optimal perioperative care. Some of the preoperative workups were spotted. Routine tests, including complete blood count (hemoglobin, white blood cells, platelets), random glucose, and albumin, are obtained before surgery and studied to see if any correlation with shunt infection or other related complications exists.

According to an observational cohort study investigating the role of some preoperative biochemical markers in predicting the risk of surgical site infection, there is evidence that low levels of hemoglobin preoperatively are significantly associated with odds of surgical site infection. A significant result was detected in 69.5% of our pediatric cases, which did not develop subsequent complications where they were found to have an average preoperative hemoglobin level [29].

Also, serum glucose is needed to be evaluated as hyperglycemia was correlated with a higher risk for surgical site infection independent from diabetes in spinal and other non-neurosurgical surgeries [30]. This was justified by the disturbance of the host immune system. It includes the inhibition of phagocytosis, complement function, and chemotaxis [30]. A significant correlation was found in our analysis showing that 60% of cases with an average preoperative glucose level did not develop shunt infection, while 73.6% did not develop shunt-related complications.

In surgical patients, it was reported that WBCs count might be associated with postoperative complications. This was manifested following endovascular interventions. However, in our study, most shunt insertion cases with normal preoperative leukocyte count did not get infected [31].

As malnourished patients are considered at a higher risk for surgical morbidity and mortality, this study investigates whether preoperative serum albumin contributes as an indicator of shunt functionality since it reflects the patient's nutritional status [32]. Therefore, albumin is evaluated in 77 cases with a mean value of 31.7 g/L. However, no relation was detected to predict if abnormal albumin levels might reflect shunt functionality.

Study limitations

This study has key limitations that should be carefully considered. Primarily, the study design is retrospective in nature. Therefore, it is subject to the information and selection biases associated with all retrospective cohort analyses. In addition, it has a mixed pediatric and adult population. We couldn't investigate some risk factors like prematurity and congenital anomalies due to insufficient patient profile data. Specifically, the rate of suspected shunt infection might be over-reported and higher than previously reported in the literature. At the same time, the definite shunt infection complication rate may be truly less than 5%. Such limitations can be addressed with future prospective studies (with standardized data collection forms) assessing the real-time incidence of shunt infection from VPS surgery.

## Conclusions

In this retrospective analysis of 132 VPS surgeries performed in 103 patients with hydrocephalus, several factors are associated with a shunt infection. This included younger than 7.5 months, with body weight less than 10 Kg, and associated with myelomeningocele. In addition, revised shunt procedures, operative time of more than 82 minutes, lack of antibiotics prophylaxis, and operating room attendees of more than four during surgery are other risk factors associated with a shunt infection. Early identification and modifications of the risk factors of shunt infection are associated with lower risks of complications and good patients outcome.

## References

[1] Del Bigio MR (1998). Biological reactions to cerebrospinal fluid shunt devices: a review of the cellular pathology. Neurosurgery.

[2] Blount JP, Campbell JA, Haines SJ (1993). Complications in ventricular cerebrospinal fluid shunting. Neurosurg Clin N Am.

[3] Maghrabi Y, Baeesa S (2017). Shunts and shunt complications.

[4] Simon TD, Hall M, Riva-Cambrin J (2009). Infection rates following initial cerebrospinal fluid shunt placement across pediatric hospitals in the United States. Clinical article. J Neurosurg Pediatr.

[5] Bierbrauer KS, Storrs BB, McLone DG, Tomita T, Dauser R (1990). A prospective, randomized study of shunt function and infections as a function of shunt placement. Pediatr Neurosurg.

[6] McGirt MJ, Zaas A, Fuchs HE, George TM, Kaye K, Sexton DJ (2003). Risk factors for pediatric ventriculoperitoneal shunt infection and predictors of infectious pathogens. Clin Infect Dis.

[7] Odio C, McCracken GH Jr, Nelson JD (1984). CSF shunt infections in pediatrics. A seven-year experience. Am J Dis Child.

[8] Bayston R, Lari J (1974). A study of the sources of infection in colonised shunts. Dev Med Child Neurol.

[9] Lee JK, Seok JY, Lee JH (2012). Incidence and risk factors of ventriculoperitoneal shunt infections in children: a study of 333 consecutive shunts in 6 years. J Korean Med Sci.

[10] Kulkarni AV, Drake JM, Lamberti-Pasculli M (2001). Cerebrospinal fluid shunt infection: a prospective study of risk factors. J Neurosurg.

[11] Apfelbaum JL, Connis RT, Nickinovich DG (2012). Practice advisory for preanesthesia evaluation: an updated report by the American Society of Anesthesiologists Task Force on Preanesthesia Evaluation. Anesthesiology.

[12] Borgbjerg BM, Gjerris F, Albeck MJ, Børgesen SE (1995). Risk of infection after cerebrospinal fluid shunt: an analysis of 884 first-time shunts. Acta Neurochir (Wien).

[13] McClinton D, Carraccio C, Englander R (2001). Predictors of ventriculoperitoneal shunt pathology. Pediatr Infect Dis J.

[14] Khan F, Shamim MS, Rehman A, Bari ME (2013). Analysis of factors affecting ventriculoperitoneal shunt survival in pediatric patients. Childs Nerv Syst.

[15] Reddy GK, Bollam P, Caldito G (2012). Ventriculoperitoneal shunt surgery and the risk of shunt infection in patients with hydrocephalus: long-term single institution experience. World Neurosurg.

[16] Vinchon M, Dhellemmes P (2006). Cerebrospinal fluid shunt infection: risk factors and long-term follow-up. Childs Nerv Syst.

[17] Ammirati M, Raimondi AJ (1987). Cerebrospinal fluid shunt infections in children. A study on the relationship between the etiology of hydrocephalus, age at the time of shunt placement, and infection rate. Childs Nerv Syst.

[18] Bokhary MA, Kamal H (2008). Ventriculo-peritoneal shunt infections in infants and children. Libyan J Med.

[19] Pople IK, Bayston R, Hayward RD (1992). Infection of cerebrospinal fluid shunts in infants: a study of etiological factors. J Neurosurg.

[20] Younger JJ, Simmons JC, Barrett FF (1987). Operative related infection rates for ventriculoperitoneal shunt procedures in a children's hospital. Infect Control.

[21] Schmidt K, Gjerris F, Osgaard O, Hvidberg EF, Kristiansen JE, Dahlerup B, Kruse-Larsen C (1985). Antibiotic prophylaxis in cerebrospinal fluid shunting: a prospective randomized trial in 152 hydrocephalic patients. Neurosurgery.

[22] Dempsey R, Rapp RP, Young B, Johnston S, Tibbs P (1988). Prophylactic parenteral antibiotics in clean neurosurgical procedures: a review. J Neurosurg.

[23] Tacconelli E, Cataldo MA, Albanese A (2008). Vancomycin versus cefazolin prophylaxis for cerebrospinal shunt placement in a hospital with a high prevalence of meticillin-resistant Staphylococcus aureus. J Hosp Infect.

[24] Kulkarni AV, Rabin D, Lamberti-Pasculli M, Drake JM (2001). Repeat cerebrospinal fluid shunt infection in children. Pediatr Neurosurg.

[25] Shannon CN, Simon TD, Reed GT, Franklin FA, Kirby RS, Kilgore ML, Wellons JC 3rd (2011). The economic impact of ventriculoperitoneal shunt failure. J Neurosurg Pediatr.

[26] Tuli S, Drake J, Lawless J, Wigg M, Lamberti-Pasculli M (2000). Risk factors for repeated cerebrospinal shunt failures in pediatric patients with hydrocephalus. J Neurosurg.

[27] Rehman AU, Rehman TU, Bashir HH, Gupta V (2010). A simple method to reduce infection of ventriculoperitoneal shunts. J Neurosurg Pediatr.

[28] Duguid JP, Wallace AT (1948). Air infection with dust liberated from clothing. Lancet.

[29] Mujagic E, Marti WR, Coslovsky M (2018). The role of preoperative blood parameters to predict the risk of surgical site infection. Am J Surg.

[30] Olsen MA, Nepple JJ, Riew KD, Lenke LG, Bridwell KH, Mayfield J, Fraser VJ (2008). Risk factors for surgical site infection following orthopaedic spinal operations. J Bone Joint Surg Am.

[31] Amaranto DJ, Wang EC, Eskandari MK, Morasch MD, Rodriguez HE, Pearce WH, Kibbe MR (2011). Normal preoperative white blood cell count is predictive of outcomes for endovascular procedures. J Vasc Surg.

[32] Dempsey DT, Mullen JL, Buzby GP (1988). The link between nutritional status and clinical outcome: can nutritional intervention modify it?. Am J Clin Nutr.

